# Protocol for a randomised trial testing a community fibrosis assessment service for patients with suspected non-alcoholic fatty liver disease: LOCal assessment and triage evaluation of non-alcoholic fatty liver disease (LOCATE-NAFLD)

**DOI:** 10.1186/s12913-020-05233-2

**Published:** 2020-04-21

**Authors:** David Brain, James O’Beirne, Ingrid J. Hickman, Elizabeth E. Powell, Patricia C. Valery, Sanjeewa Kularatna, Ruth Tulleners, Alison Farrington, Leigh Horsfall, Adrian Barnett

**Affiliations:** 1 Australian Centre for Health Services Innovation (AusHSI), Queensland University of Technology, GPO Box 2434, Brisbane, QLD 4001 Australia; 2 University of the Sunshine Coast, Locked bag 4, Maroochydore DC, QLD 4558 Australia; 3 The University of Queensland, St Lucia, QLD 4072 Australia; 4 Department of Nutrition and Dietetics, Princess Alexandra Hospital, Brisbane, QLD 4102 Australia; 5 Centre for Liver Disease Research, Faculty of Medicine, Translational Research Institute, The University of Queensland, Brisbane, QLD Australia; 6 Department of Gastroenterology and Hepatology, Princess Alexandra Hospital, Brisbane, QLD 4102 Australia; 7 QIMR Berghofer Medical Research Institute, Locked bag 2000, Royal Brisbane Hospital, QLD 4029 Australia

**Keywords:** Non-alcoholic fatty liver disease, NAFLD, Community-based management, Protocol, Randomised controlled trials, Economic evaluation, Implementation evaluation

## Abstract

**Background:**

Non-alcoholic fatty liver disease (NAFLD) is the most common type of chronic liver disease in Australia and its recent increase mirrors the obesity and type 2 diabetes epidemics. Currently, many patients who present to primary care with abnormal liver function tests or steatosis on liver ultrasound are referred for assessment in secondary care. Due to the large number of patients with NAFLD, this results in long waits for clinical and fibrosis assessment, placing unnecessary burden on the public hospital system.

**Methods:**

We will conduct a 1:1 parallel randomised trial to compare two alternative models of care for NAFLD. Participants will be randomised to usual care or the LOCal Assessment and Triage Evaluation (LOCATE) model of care and followed for 1 year. We will recruit patients from the non-neighbouring Sunshine Coast and Metro South Hospital and Health Services (HHSs) in Queensland, Australia. Our primary outcome of interest is time to diagnosis of high-risk NAFLD, based on the number of participants in each arm of the study who receive a diagnosis of clinically significant fibrosis. Two hundred and 34 participants will give us a 95% power to detect a 50% reduction in the primary outcome of time to diagnosis of high-risk disease. We will also conduct an economic evaluation, evaluating the cost-effectiveness of the new model of care. We will also evaluate the implementation of the new model of care.

**Discussion:**

It is anticipated that the results of this study will provide valuable new information regarding the management of NAFLD in the Australian setting. A relatively simple change to care could result in earlier identification of patients with significant liver disease and lower overall costs for the health system. Results will be directly disseminated to key staff for further distribution to consumers, policy- and decision-makers in the form of evidence briefs, plain language summaries and policy recommendations.

**Trial registration:**

The trial was registered on 30 January, 2020 and can be found via ANZCTR - number ACTRN12620000158965.

## Background

Non-alcoholic fatty liver disease (NAFLD) is the most common type of chronic liver disease in Australia and its recent increase mirrors the obesity and type 2 diabetes epidemics [1, 2]. The prevalence of disease in Australia is approximately 30% of adults, comparable to other developed countries [3]. Currently, many patients who present to primary care with abnormal liver function tests or steatosis on liver ultrasound are referred for assessment in secondary care. Due to the large number of patients with NAFLD, this results in long waits for clinical and fibrosis assessment, placing unnecessary burden on the public hospital system. Depending on the presence of risk factors such as diabetes, 60 to 90% of patients with NAFLD do not have advanced fibrosis, are not at risk of chronic liver disease complications, and can be safely managed in primary care. At the other end of the risk scale, recent community-based studies have suggested that as many as 12 to 17% of primary care patients with NAFLD and type 2 diabetes may have clinically significant liver disease [4, 5]. The most important predictor of liver-related mortality is the extent of fibrosis – once NAFLD is identified, assessment of risk stratification is crucial to make decisions about management and referral. Due to the lack of risk stratification at primary care level, coupled with poor general practitioner knowledge about NAFLD [6], many patients with advanced disease may not undergo timely referral into secondary care. This exposes them to the risk of disease progression, decompensation and avoidable hospital admissions [7].

The predicted increases in NAFLD in the next decade will create demand on health services and have potentially large economic costs. As the number of NAFLD cases increase, the health system will incur increased costs associated with its diagnosis, management, and disease progression [8]. The estimated annual cost of liver diseases in Australia is $51 billion, of which NAFLD accounts for a considerable share [9], although few studies have reported economic outcomes [10].

NAFLD reduces health-related quality of life, particularly for those with severe disease who have higher complication risks. We propose that a community fibrosis assessment service will provide NAFLD patients with an integrated model of care, providing accurate stratification of disease risk, timely identification of advanced disease, and guidance for general practitioners (GPs) to enable better community management of mild disease. Gathering evidence from a prospective trial will address critical knowledge gaps in the Australian evidence-base and enhance the likelihood that the new findings will be implemented into routine practice.

### Hypothesis

The new model of care for NAFLD patients with fibrosis risk stratification of patients in the community will improve patient outcomes and be cost-effective, by more appropriately triaging NAFLD patients, reducing time to appropriate care, and avoiding unnecessary hospital appointments.

## Methods

### Study design

#### Parallel randomised trial

We will conduct a 1:1 parallel randomised trial to compare two alternative models of care for NAFLD. Participants will be randomised to usual care or the LOCal Assessment and Triage Evaluation (LOCATE) model of care and followed for 1 year to monitor their outcomes.

#### Reasoning

A parallel randomised trial is an excellent study design for estimating the benefits of a new model of care compared with a usual model of care. Randomising groups in parallel means that variables that change over time cannot confound the comparison of interest, because any changes are equally experienced by both groups.

#### Limitations

Randomised trials sometimes do not reflect real world settings because of their inclusion and exclusion criteria [11]. We aim to use as similar a patient group in the trial as would be eligible for the service in real life.

### Study setting

We will recruit patients from the Sunshine Coast and Metro South Hospital and Health Services (HHSs). These are non-neighbouring HHSs located in the south-east of Queensland. Combined, these HHSs provide healthcare to approximately 30% of Queensland’s population (1.4 M residents).

#### Potential for risk, burdens and benefits to participants

There is little risk for participants as the study involves either current usual care or a modification to a model of care that uses an established modality and treatment pathway. The study makes extensive use of routinely collected data, which reduces the burden on participants. Participants in the LOCATE model of care group may benefit from a faster diagnosis, as per our key study hypothesis.

#### Discontinuation of whole study

The study will only be discontinued if a regulatory body, funding body, or Human Research Ethics Committee (HREC) judges it necessary for medical, safety, regulatory, or other reasons consistent with applicable laws, regulations and good clinical practice.

### Study population

#### Patient inclusion criteria

Patients will be included if they:

- Have had NAFLD diagnosed or suspected by their GP.
- Are aged 18 years or over.
- Understand the consent procedures and give their full consent.
- Consent to access of their data from Queensland Health and Medicare Benefits Schedule (MBS) and Pharmaceutical Benefits Scheme (PBS).

#### Patient exclusion criteria

Patients will be excluded if they:

- Are pregnant.
- Have advanced cardiac disease or another terminal illness.
- Have high current alcohol consumption, defined as two or more standard drinks per day.
- Have Hepatitis B or C.
- Require priority review at the Hepatology Clinic, for instance, if liver imaging suggests cirrhosis or a focal liver lesion.
- Have been evaluated in a specialist hepatology clinic in the previous 12 months.
- Have plans to leave the area within the next 12 months.

The exclusion criteria are designed to reduce loss to follow-up and exclude patients who would be ineligible for a wider roll out of the service, or who require priority care and review.

### Aims

#### Primary clinical outcome

The time to diagnosis of high-risk NAFLD will be calculated based on the number of participants in each arm who receive a diagnosis of clinically significant fibrosis (transient elastography (TE) over 8.0 kPa) [7], with the time measured from date of GP referral to a confirmed diagnosis.

#### Secondary clinical outcomes

Secondary clinical outcomes of interest are:

- Time to first successful fibrosis assessment with Fibroscan
- Reduction in hospital admissions
- Reduction in emergency department presentations
- Reduced time to additional screening/testing for liver disease
- Improved health-related quality of life
- Reduced hepatocellular carcinoma (HCC) detected outside specific surveillance
- Reduced variceal bleeding occurring without variceal surveillance
- Increased statin use
- Increased referrals to a specialist, other than a hepatologist, for example, to a dietician or exercise physiologist
- Reduced mortality

#### Primary economic outcome

To evaluate the cost-effectiveness of the new model of care, where the costs and health outcomes associated with the LOCATE model of care are compared to those experienced under usual care.

#### Secondary economic outcome

We will model the potential for longer term cost-savings due to improved identification and stratification of high-risk NAFLD patients in the community and the reduction in high cost, hospital-based complications.

#### Implementation outcomes

To evaluate the implementation of the new model of care we will:

- Map the reach of the model of care.
- Determine the impact of the model of care on the proportion of low risk and higher risk NAFLD being seen in specialist clinics.
- Determine if patients with low risk and high risk NAFLD received appropriate care for their condition.
- Determine the factors associated with implementation of the model of care including: (a) individual factors, (b) institutional factors and (c) systemic factors.
- Explore the GP and patient experience with the LOCATE model of care including acceptability of community-based screening and care for lower risk conditions.
- Determine whether LOCATE is a sustainable and scalable model of care in the community.

### Description of intervention and comparator

**Fig. 1:**
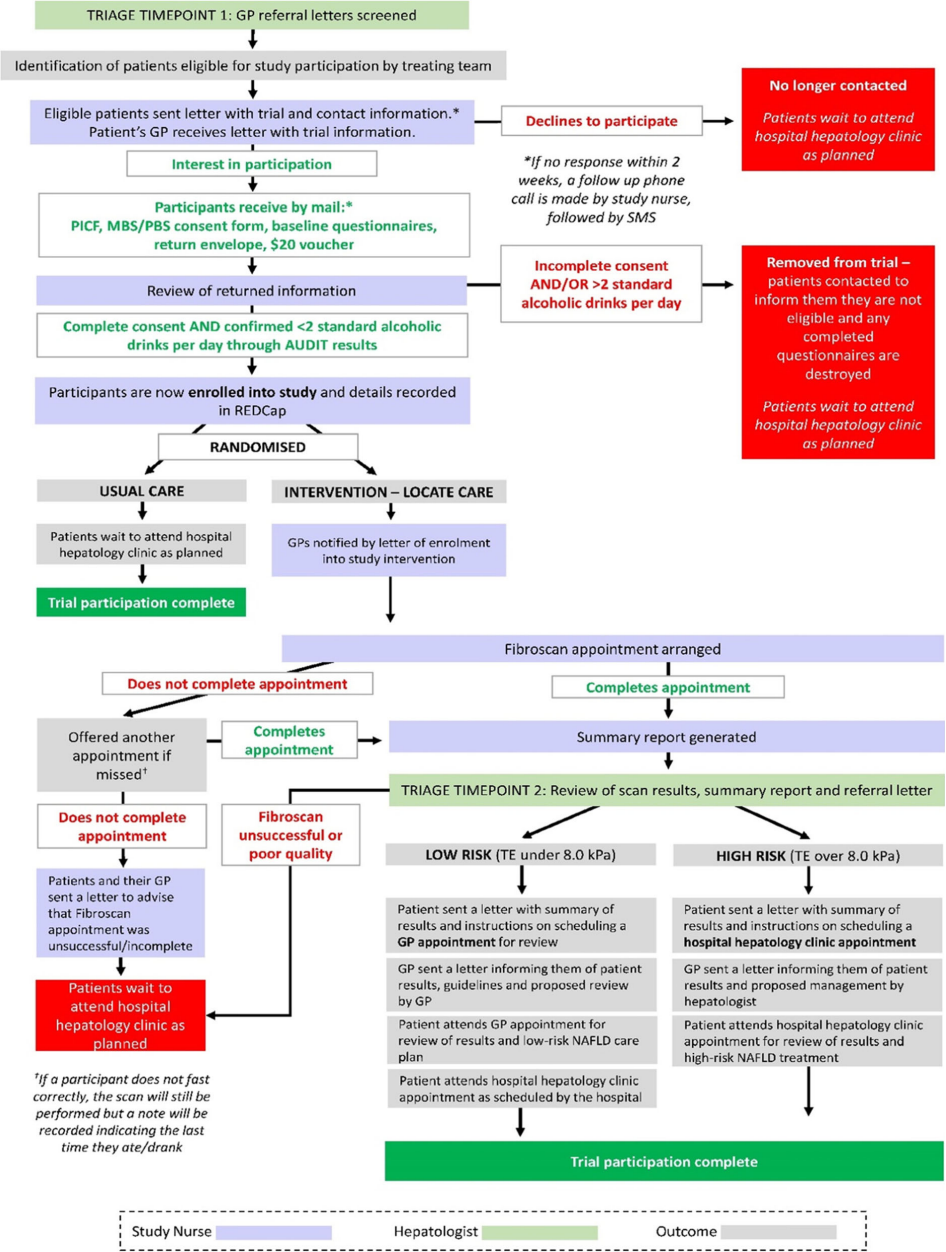
Recruitment and patient flow pathway for LOCATE-NAFLD study

Participant progress through the study is shown in Fig. 1. The starting event is a patient visiting their GP where if there are enough concerns about the patient’s liver health, the GP will write a referral requesting a specialist hepatology clinic review/appointment. Referral letters will be screened by a hepatologist at the Sunshine Coast University Hospital and Logan Hospital hepatology clinics to identify potentially eligible patients.

#### Randomised to LOCATE

Study participants randomised to the new model of care will be invited to attend a local clinic to have their suspected NAFLD assessed with mobile transient elastography, using the Fibroscan machine. The invitation and assessment will be made by a specialist study nurse, who will write a report for the hepatologist. The hepatologist will triage patients to low- or high-risk, depending on the assessment of liver scarring and potentially other clinical indicators.

##### Low risk

Participants with a low Fibroscan score - TE under 8.0 kiloPascal (kPa) - will be classified as ‘Low Risk’. A letter will be sent to participants with a summary of their results and instructions on scheduling a GP appointment for review. A letter will be sent to the referring GP informing them of the results and with advice on multidisciplinary NAFLD management and guidelines for follow up [6].

These patients will remain on the waitlist to see a hepatologist at the hospital clinic as originally intended.

##### High risk

Participants with clinically significant fibrosis - TE over 8.0 kPa - will be classified as ‘High Risk’. Participants will be sent a letter summarising their test results and asking them to call the hospital hepatology clinic to arrange an appointment. GPs will be sent a letter informing them of the results and proposed management.

#### Randomised to usual care (comparator)

For those participants randomised to usual care, their referral letters will be dealt with in the usual way, and the patients will wait to see a hepatologist at the hospital clinic as originally intended.

#### Fibroscan service

The Fibroscan service will enable participants to undergo reliable assessment of the severity of liver fibrosis and to identify those with advanced fibrosis and cirrhosis who need both secondary care input and surveillance for liver cancer. The mobile Fibroscan clinics will occur on a rotational basis in the two regions using GP clinics and primary care facilities.

The Fibroscan will give the following test results:

- Liver stiffness measurement - a marker of clinically significant fibrosis or advanced cirrhosis.
- Controlled attenuation parameter, to detect and quantify steatosis [12].

Examinations will be performed by a trained clinical nurse with extensive Fibroscan experience. Using an experienced operator is very important for producing reliable scans. Participants who did not attend an arranged appointment will be offered another appointment.

Around 7% of Fibroscans performed do not provide a valid reading. This is multifactorial, including issues such as skin to capsule depth, body habitus and rib spacing. These participants will be scheduled for a hepatology clinic visit as per the usual model of care. We will record the number of times the scan was unsuccessful and the reason why.

#### Other diagnostic information

Some additional information may be collected from GP referral letters if it is available. This information, including blood tests, may be used to help in triage and diagnosis, and may include:

- Fibrosis-4 score.
- NAFLD fibrosis score [7].
- Enhanced Liver Fibrosis

### Description of all processes

#### Data collection

#### Referral letter

Date of referral, age, BMI, presence of type 2 diabetes and relevant blood tests such as AST, ALT, albumin, platelet count, viral serology, ferritin and transferrin saturation will be extracted from the referral letter.

#### Study questionnaire

The study recruitment questionnaire will contain:

- Year of birth for calculating age.
- Gender.
- Height and weight for calculating BMI.
- Alcohol consumption in the past year using the 10-item screening tool, Alcohol Use Disorders Identification Test (AUDIT) [13].
- Current employment status for the economic analysis.
- EQ-5D-3 L [14] for estimation of health-related quality of life for the economic analysis.

#### Twelve-month telephone follow-up

Participants will be telephoned by study staff at 12 months post recruitment and asked:

- EQ-5D-3 L questions for quality of life.
- Current employment status.

#### Twelve-month chart audit

Study nurses will audit the patients’ individual medical record at 12 months post-recruitment and extract much of the data needed for the primary and secondary outcomes.

#### Data management

#### Data sources

Data for outcomes involving contact with tertiary care will be collected from Queensland Health administrative databases.

For the economic evaluation, a state-based model will be developed. Costing data will be prospectively collected from multiple sources to measure costs associated with; hospital utilisation, pharmaceutical and primary care use, and patient travel costs, lost productivity and other out-of-pocket expenses. To estimate health utility, quality of life data will be collected using the EuroQol EQ-5D-3 L tool and assigned to each health state in the model. Data will be collected from participants at two time points, as previously described.

For the implementation objectives, we will audit data collected throughout the study, and conduct semi-structured interviews with key stakeholders and consumers. These interviews will occur within 8 weeks of the trial’s conclusion.

#### Participant data

Following recruitment and the return of the consent forms and baseline questionnaires to the study team, a research assistant will enter the data directly into the secure REDCap platform and determine whether participants meet the eligibility requirements according to the inclusion/exclusion criteria. The 12-month telephone survey will be entered directly into REDCap by the study staff.

#### Data storage

The project manager, investigators and other project staff are responsible for maintaining a comprehensive and centralised bibliographic filing system of all study-related, essential documentation, suitable for inspection at any time by the approving HREC or applicable regulatory authorities.

A detailed data management plan will be completed and will ensure that all document data will be stored on hard disk drives. These computers will be networked to a file storage server, where an automated batch file copy procedure will back up the entire hard disk drive of each computer daily.

#### Data retention

Study records will be retained as per the Queensland Government University Sector Retention and Disposal Schedule. At the end of the study, final, non-identifiable data sets will be deposited in QUT’s Research Data Storage System. In line with publication embargoes and requirements, we will generate a document object identifier for each non-identifiable data set and make this record publicly accessible.

#### Data access

Processes for data access will be established in the data management plan. During the study, only members of the study team will access patient data.

#### Recruitment and consent

#### Recruitment

Potential participants will be identified based on the referral letter sent from their GP to the hepatology clinic. Potentially eligible participants will be sent a letter containing an invitation to participate and the contact details of a study nurse.

NEGATIVE OR NO RESPONSE: If no response to the initial letter is received after 2 weeks, a study nurse will contact the patient by phone. If the potential participant does not answer the phone, an SMS will be sent from a study phone to their mobile, informing them that it was a study nurse who called, and providing a brief explanation of the study and contact information. If at any stage of this process they state they are not interested, they will not be contacted again.

POSITIVE RESPONSE: Those interested in participating will be able to call a study nurse to discuss the trial. Interested participants will then be mailed the Participant Information and Consent Form, MBS/PBS consent forms, withdrawal form, study questionnaires, a $20 voucher and a reply-paid return envelope.

#### Consent

Potential participants who agree to be contacted after the recruitment process will be sent the study forms in the mail and asked to read, sign and return the forms using the reply-paid envelope. The study nurses will make one follow-up call, and SMS if not answered, to potential participants who have not returned the consent and enrolment questionnaires after 2 weeks. We will record the number of participants declining to consent and will also record the number of potential participants who could not be contacted.

#### Participant withdrawal

Participants will be able to withdraw with no negative consequences from further participation at any time, either in writing using the form provided to them at recruitment, or verbally to either a study team member and/or chief investigator. We will not collect any further data from the participant but will keep the data already collected unless the participant makes it clear that they do not wish this.

#### Randomisation

A 1:1 randomisation list will be created by the study statistician in the *R* software. The list will be stratified by health service (Sunshine Coast and Metro South) and will be in randomised blocks of size 6 and 8. This creates balanced groups over time and means that group sizes will be approximately equal if recruitment ends early. The list will be uploaded into the REDCap software [15]. Patients will be randomised once they have returned the signed consent form and questionnaires.

#### Blinding

It is challenging to hide the randomised group because this is an open-label study where both the participants and researchers will be aware of what group participants are in. Baseline data will be collected blind to randomised group, as it will be done prior to randomisation.

#### Safety evaluations

The following will be used to evaluate the safety of staff and patients involved in the study:

- protocol deviation and adverse events reporting.
- incident and unanticipated problem monitoring.

#### Protocol deviations and adverse event reporting

The project manager is responsible for ensuring that all protocol deviations and adverse events are reported to the approving HREC and site Research Governance Officers. A protocol deviation is any noncompliance with the study protocol or HREC requirements and may be either on the part of the participant, the investigator, project team or the study site staff. Corrective actions will be implemented promptly.

#### Incident and unanticipated problem monitoring and reporting

The project manager is responsible for ensuring that all incidents and unanticipated problems observed by the investigator/s, project team or reported by sites are collected, reviewed and recorded in the source documents.

#### Monitoring

##### Data monitoring committee

We will recruit an independent study monitoring committee of three researchers - a clinician, a statistician, and one trialist - who have not collaborated with the chief investigator team in the past 5 years. This group will have controlled access to the study’s REDCap data collection site, where they will be able to view live reports on recruitment but not be able to see individual patient information. Recruitment reports show the current cumulative sample size and summary statistics on the sample’s baseline characteristics. We will prompt the data monitoring committee for 6-monthly feedback, share a one-page report on our progress against the milestones, and share this protocol with them.

### Statistical analysis

#### Sample size and statistical power

We have a minimum total sample size of 156 participants for our primary outcome of time to diagnosis, but we will aim for a sample size of 234. Two hundred and 34 participants will give us a 95% power to detect a 50% reduction in the primary outcome of time to diagnosis of high-risk disease. Our lower sample size of 156 gives us an 83% power. We used a two-sided 5% significance level. We assumed an average diagnosis time of 180 days in the usual care group, and a halving of this time in the intervention group. We assumed that 5% of participants would be censored due to death or loss to follow-up. The sample size was estimated by simulating survival data using the “sim.survdata” function from the “coxed” package in *R* using 1000 simulations [16]. We assumed a log-normal hazard function with a standard deviation of 10 days. The statistical code to run the sample size is available here: https://github.com/agbarnett/LOCATE. We assume that half of all patients approached will agree to participate, hence, we aim to approach 468 people.

#### Data analysis – overall considerations

We will present results as means and 95% confidence intervals. We will include *p*-values in our reports, but will aim not to present them in any external reports or papers given the widespread misunderstanding of their meaning [17]. We will use residual checks for all our models and will look for non-normality and outliers. These checks will be published as a supplementary to any paper and on *github* (https://github.com/agbarnett/LOCATE).

#### Per protocol definition

We will use an intention-to-treat approach meaning that participants will be analysed according to their randomised group, regardless of whether they followed that model of care [18]. In a sensitivity analysis we will use a per protocol analysis by only including patients that attended their Fibroscan appointment and had a successful scan. The per protocol analysis will be applied to the primary and secondary outcomes, and the economic analysis.

#### Analysis of the primary outcome

We will use survival analysis methods to examine the primary outcome of time to diagnosis of high-risk NAFLD. We will use Kaplan–Meier plots to highlight any differences between the usual care and intervention groups. We will use parametric Weibull survival models to examine a statistical difference. We will check the parametric assumption and the model residuals.

#### Analyses of secondary outcomes

For the three time-to-event outcomes of NAFLD-associated admissions and re-presentations, and first fibrosis assessment, we will use Kaplan–Meier plots and Weibull survival models as per the primary outcome.

For health-related quality of life we will model the participants’ 12-month result whilst adjusting for their baseline, which is the equivalent to examining the within-participant change in quality of life and helps adjust for regression to the mean [19, 20]. We will use a linear regression model and check the model residuals.

Statin use will be compared between groups by examining the number of participants given a new statin script during the follow-up period. The denominator will be the number of patients not on statins at the time of their referral letter to the hepatology clinic. We will compare the numbers using a 2-by-2 table and Chi-squared test. Deaths and HCC detection will be compared between groups using a 2-by-2 table. As these two outcomes are likely to be rare, we anticipate needing to use Fisher’s exact test to look for statistical differences between the two groups.

#### Missing data

We will report the number and percent of missing data for every study variable. We will investigate variables that have relatively high levels of missing data (over 5%) and seek to identify the cause of the missing data and the potential for bias.

#### Interim analysis

There is no planned interim analysis nor any stopping rules.

#### Planned subgroup and adjusted analysis

There are three planned subgroup or adjusted analyses:

1. The per protocol analysis.
2. Sensitivity analysis for missing data.
3. Separate analyses according to age cohorts (younger - under 75) and (older - 75 and over).

#### Additional information

The results will be written up using the Consolidated Standards of Reporting Trials (CONSORT) and Template for Intervention Description and Replication (TIDieR) checklists [21, 22]. As per the CONSORT guidelines, we will not use statistical tests to compare the two groups of patients at baseline and will instead look for differences using a table of summary statistics.

## Discussion

### Dissemination of results

Results will be directly disseminated to each participating health service using a report that will include:

- A brief lay summary of the results in their health service.
- The detailed overall results.

At recruitment all participants will be asked if they would like to receive a summary of the results, and those who agree will be emailed a one-page lay summary once the analyses have been written up.

#### Dissemination of results to health consumers, policy makers and other stakeholders

Results will be directly disseminated to key staff in Queensland Health for further distribution to consumers, policy- and decision-makers in the form of evidence briefs, plain language summaries and policy recommendations. A publication plan that details the likely papers and conferences will be established to inform systematic publication of results through the clinical and academic communities. We will adhere to the International Committee of Medical Journal Editors requirements for authorship and will report the contributions of each author. We will not use professional writers. The protocol will be publicly available via ANZCTR (https://www.anzctr.org.au) – trial number ACTRN12620000158965. This is a nationally important health services research trial that examines a change to the model of care for triaging patients with liver disease. A relatively simple change to care could result in earlier identification of patients with significant liver disease and lower overall costs for the health system. The study uses a strong randomised design and will collect high quality data to provide clear information for decision-makers. The implementation component of the study will provide valuable information for other health services that may want to implement the model of care based on the trial results.

## Data Availability

The datasets generated and/or analysed during the current study are available in the *github* repository (https://github.com/agbarnett/LOCATE).

## Abbreviations

LOCATE-NAFLD: LOCal Assessment and Triage Evaluation of Non-Alcoholic Fatty Liver Disease
LOCATE: LOCal Assessment and Triage Evaluation
NAFLD: Non-alcoholic fatty liver disease
HHSs: Hospital and Health Services
ANZCTR: Australian New Zealand Clinical Trials Registry
MBS: Medicare Benefits Schedule
PBS: Pharmaceutical Benefits Scheme
CONSORT: Consolidated Standards of Reporting Trials
TIDieR: Template for Intervention Description and Replication
HCC: Hepatocellular Carcinoma
GP(s): General Practitioner(s)
BMI: Body Mass Index
AST: Aspartate aminotransferase
ALT: Alanine transaminase
AUDIT: Alcohol Use Disorders Identification Test
TE: Transient Elastography
HREC: Human Research Ethics Committee
SMS: Short Messaging Service
